# Pulmonary Telerehabilitation for People With Chronic Obstructive Pulmonary Disease in Brazil: A Mixed-Methods Feasibility Study

**DOI:** 10.5195/ijt.2023.6555

**Published:** 2023-05-11

**Authors:** Luis H. G. Neves, Carla Malaguti, Marissa R. Santos, Laura A. Cabral, Laura B.D. da Silva, Hugo H. de Oliveira, Alessa S. S. Brugiolo, Anderson José, Anne E. Holland, Cristino C. Oliveira

**Affiliations:** 1 Post-Graduate Research Program on Rehabilitation Sciences and Physical Function Performance, Faculty of Physiotherapy, Federal University of Juiz de Fora, Governador Valadares, Minas Gerais, Brazil; 2 Post-Graduate Research Program on Rehabilitation Sciences, School of Physical Education, Physical Therapy, and Occupational Therapy. Department of Physical Therapy, Federal University of Minas Gerais, Belo Horizonte, Brazil.; 3 Department of Physiotherapy, Alfred Health, Melbourne, Victoria, Australia.; 4 Institute for Breathing and Sleep, Heidelberg, Victoria, Australia.; 5 Central Clinical School, Monash University, Melbourne, Victoria, Australia.

**Keywords:** Chronic Obstructive Pulmonary Disease, Feasibility studies, Telerehabilitation

## Abstract

This study assessed the feasibility of pulmonary telerehabilitation‧s (PTR) acceptability, implementation, practicality, and adaptation for people with Chronic Obstructive Pulmonary Disease (COPD) in Brazil. It also explored associations with clinical and socioeconomic features of Brazilians with COPD. This mixed-method study included thirty-one participants with COPD (age 62±10 years; FEV_1_= 72±14% predicted). Most participants (74.2%) reported good PTR session acceptability on the System Usability Scale and scores of 4.6±0.3 and 4.5±0.6 on a 1–5 Likert-type scale of implementation and practicality, respectively. Participants suggested adaptations for better comfort on the exercise bike and varying exercise modalities. PTR acceptability was associated with participants' younger age (r_s_=−0.57, p<0.01) and higher education (r_s_=0.51, p<0.01). PTR is feasible for people with COPD in Brazil regarding acceptability, implementation, practicality, and adaptation. Younger age and higher educational level are associated with greater PTR acceptability.

Chronic Obstructive Pulmonary Disease (COPD) is a progressive lung disease related to increased morbidity and mortality (Global Initiative for Chronic Obstructive Lung Disease [GOLD], 2022). COPD is among the three most prevalent causes of death worldwide, and 90% of these deaths occur in low- and middle-income countries (Halpin et al., 2019) with relevant social and economic burdens (GOLD, 2022). COPD affects the respiratory system and has systemic manifestations related to nutritional depletion, increased fatigue, sarcopenia, and impaired exercise capacity (Agustí et al., 2003). There is robust evidence for non-pharmacological treatments for COPD; these include pulmonary rehabilitation, education, self-management, and integrative care (GOLD, 2022; Lacasse et al., 2015).

The benefits of pulmonary rehabilitation in people with COPD are well documented and include decreased dyspnea, anxiety, and depression symptoms; it also improves exercise tolerance, muscle strength, and quality of life (Spruit et al., 2013). Despite clear recommendations, pulmonary rehabilitation has limited availability and is often inaccessible (Rochester et al., 2015). In high-income countries, the national capacity of pulmonary rehabilitation per annum is critical, available to ≤1.2% of the estimated COPD population (Desveaux et al., 2014). Critical barriers to program participation also include reduced availability of rehabilitation centres, inaccessible locations, inadequate referrals, increased costs, and transportation constraints (Cox et al., 2017), particularly in low- and middle-income countries where complex factors influence the successful implementation of interventions targeting chronic respiratory diseases (Brakema et al., 2020). People living with COPD in socioeconomically deprived areas are less likely to complete rehabilitation programs than their peers in the least deprived areas (Steiner et al., 2017). The COVID-19 pandemic amplified the health inequalities in this population (Gardiner et al., 2022).

Pulmonary telerehabilitation (PTR) programs using information and communication technologies have been included in managing COPD to provide home-based treatment or deliver specialized care remotely in rehabilitation centres (Cox et al., 2021). A recent global scenario of social isolation during the COVID-19 pandemic reinforced the importance of rehabilitation strategies to maintain care for those with chronic respiratory diseases (Hollander et al., 2020). In Brazil, the use of technology has increased in recent years, with 69.3% of households covered by internet access, particularly smartphones (97.2%), which facilitate the delivery of PTR (Instituto Brasileiro de Geografia e Estatística [IBGE], 2018). Although low- and middle-income countries are disproportionately burdened by COPD, current evidence of PTR feasibility is mainly based on studies from high-income countries (Bedra et al., 2013; Holland et al., 2013; Liacos et al., 2018; Paneroni et al., 2015; Sönnerfors et al., 2020; Zanaboni et al, 2013). Extrapolating the evidence from high-income countries may be inappropriate because of health, economic, and cultural differences. The PTR feasibility aspects in Brazil as a middle-income country are yet to be investigated.

The primary aim of this study was to evaluate the feasibility of an attainable simulated PTR session for people with clinically stable COPD in Brazil regarding aspects of acceptance, implementation, practicality, and adaptation. The secondary aim was to explore the association between the PTR acceptability and clinical and socioeconomic features of Brazilians with COPD.

## Methods

### Study Design and Ethics

This cross-sectional mixed-methods feasibility study followed the Good Reporting of A Mixed Methods Study (GRAMMS) (O'Cathain et al., 2008). The four feasibility aspects studied in a PTR laboratory-simulated session were: acceptability, implementation, practicality, and adaptation (Bowen et al., 2009). Data were collected from November 2020 to October 2021 at the Physiotherapy Clinical School at the Federal University of Juiz de Fora, Governador Valadares, Brazil, following institutional preventive biosafety measures against COVID-19. Approval was sought from the University Human Research Ethics Committee (#11531019.9.0000.5147), and written informed consent was obtained from all participants.

## Participants

Thirty-one participants were recruited, consistent with the minimum recommended for adequate content analysis using a qualitative approach (Mason, 2010). Participants were recruited from public and private primary health care and specialized respiratory medicine services. The inclusion criteria were COPD diagnosis confirmed by spirometry according to GOLD (GOLD, 2022), being clinically stable in the last 30 days, ability to perform a simulated laboratory-based PTR session and understanding of measures used. The exclusion criteria were a COPD acute exacerbation in the previous four weeks, recent chest, abdominal, or other major surgeries, or any clinical contraindications to perform or attend the study assessments.

### Procedures

During the first visit, participants were interviewed for COPD clinical and socioeconomic characteristics, assessed for secondary outcomes (described below), and attended an education session on PTR equipment use. During the education session, participants were instructed to turn on and unlock the smartphone connected to the wi-fi internet (Asus Zenfone 5, ASUSTeK Computer Inc., Peitou, Taipei, Taiwan), open the commercially available and user-friendly Whereby.com videoconferencing application (Video Communication Services AS©, Måløy, Norway), and enter the virtual room. The application was logged in advance in the virtual room for easy and direct access with encrypted transmitted data. Dumbbells, elastic bands, the strength exercise illustration cards, and the finger pulse oximeter (G-Tech Oled Graph, Beijing Choice ElectronicTechnology Co., Ltd, Beijing, P.R. China) were positioned on a side table near the exercise bike (O'Neal TP 320, Deluxe Magnetic Horizontal Bike, Camarillo, CA, USA). During the videoconferencing, instructions were also provided on how to show the oximeter display, oxygen saturation, and heart rate monitoring to the therapist. Two adjustable stands were attached to the modified Borg dyspnea scale (0–10) (Borg, 1982) and the smartphone to the exercise bike. Any participant's query related to equipment use was addressed during the first visit to ensure that the participant could independently manage the PTR devices, including the internet-enabled smartphone and the exercise equipment, without any in-person help.

The laboratory-based PTR session was performed on the second visit, followed by questionnaires and a structured interview to assess feasibility. A 40-min PTR session was conducted individually using two separate rooms without communication, one room for the simulated PTR session and the other for the physiotherapist's telemonitoring. The training physiotherapist supervised the PTR session from the telemonitoring room. A second research physiotherapist stayed in the PTR room for participants' safety purposes only, with no interaction with the participant. The exercise program was based on the Lung Foundation Australia Pulmonary Rehabilitation Toolkit (Lung Foundation Australia, 2020). The exercise training component of the simulated pulmonary rehabilitation session included a cycling exercise period of 20 minutes and supervised resistance training for upper and lower limbs using dumbbells of 0.5 kg or elastic bands, with strength exercises described and illustrated on exercise cards. An *a priori* individualized exercise prescription was not carried-out as a simulated session. The reasons for the session interruption and the number of adverse events were recorded.

### Primary Outcomes

Four aspects of feasibility were studied: acceptability, implementation, practicality, and adaptation. Acceptability was assessed using the System Usability Scale (SUS), a 10-item questionnaire, which generates an index of user satisfaction from 0 to 100; higher scores indicate better usability and good acceptability (Jordan & Al 1996; Martins et al., 2015). A score above 68 is considered ‘above average’ acceptability (Martins et al., 2015). The SUS is reliable and correlates with usability measures for varying communication systems (Klug, 2017). The aspects of implementation and practicality were assessed using statements adapted from previous feasibility studies of PTR in individuals with COPD (Bedra et al., 2013; Paneroni et al., 2015). Implementation statements included information like: ‘This device would help me exercise at home if I had it available’ or ‘ I would have a specific place available for installing and using these devices in my home,’ whereas practicality information included statements like: ‘The time spent in the exercise session was adequate’ or ‘It was easy to use the exercise bike.’ Participants were asked to score each statement on a 5-item Likert-type scale from 1: totally disagree to 5: totally agree. Finally, the adaptation aspect was assessed using a structured interview including the following questions: ‘In your opinion, is there anything else that could improve the delivery of rehabilitation that was not available during this session?’ and ‘Do you have any suggestions for improving the devices used, which could improve your experience in this session?’ A researcher not involved in the study conceptualization conducted the interviews. The interviews were audio-recorded and transcribed in full for content analysis. Qualitative data were reported following the Standards for Reporting of Qualitative Research (O'Brien et al., 2014).

### Secondary Outcomes

Spirometry was performed according to the American Thoracic Society and the European Respiratory Society recommendations (Graham et al., 2019). The Spirobank II Spirometer (Medical International Research, Rome, Italy) was used, and reference values were applied (Sociedade Brasileira de Pneumologia e Tisiologia, 2002). The non-fully reversible airflow limitation (Forced Expiratory Volume in 1 second/Forced Vital Capacity [FEV1/FVC] < 0.7 post-bronchodilation) recorded by spirometry confirmed the diagnosis of COPD. The airflow limitation severity was based on the postbronchodilator value of FEV1 (% reference) as follows: GOLD 1, mild: FEV1 ≥80% predicted; GOLD 2, moderate: 50% ≤ FEV1 <80% predicted; GOLD 3, severe: 30% ≤ FEV1 <50% predicted; and GOLD 4, very severe: FEV1 <30% predicted. The Modified Medical Research Council dyspnea scale (mMRC) was used to assess physical restrictions in daily living due to dyspnea (Mahler & Wells, 1988). The mMRC consists of five statements that grade dyspnea from none (Grade 0: ‘I only get breathless with strenuous exercise’) to almost complete incapacity (Grade 4: ‘I am too breathless to leave the house or I am breathless when dressing’). The COPD Assessment Test (CAT) questionnaire was applied to determine the COPD clinical burden. The CAT is a short 8-item comprehensive multidimensional questionnaire with scores ranging from 0 to 40 and higher scores representing worse health status. The CAT has excellent interobserver and intraobserver reliability and is associated with clinical outcomes in COPD (da Silva et al., 2013).

Physical activity was assessed using the Actigraph GT3X^©^ accelerometer (Actigraph LLC, Pensacola, FL, USA), an objective, valid and accurate measure of physical activity in COPD (Byrom & Rowe, 2016). The accelerometer was provided at the end of the first assessment visit, and physical activity was measured before the PTR simulated session on the second visit. Participants were instructed to wear the device on the waist above their right leg for ten consecutive days during daily activities, except during sleep, bathing, or water activities. A diary was provided to record usage time or register adverse events. Time spent in sedentary, light, moderate, vigorous, and very vigorous activities and the number of steps were obtained through activity count and acceleration. Cut-off points determined the physical activity level based on the counts per minute (CPM) registered (Freedson et al., 1998): Sedentary: 0–99 CPM, Light: 100–1951 CPM, Moderate: 1952–5724 CPM, Vigorous: 5725–9498 CPM, and Very Vigorous: > 9499 CPM. Data obtained on the first and last day of use were excluded. At least five days with ten hours or more of recording were considered suitable for analysis (Byrom & Rowe, 2016).

The economic status was assessed using the Critério Brasil from the Brazilian Association of Research Companies (ABEP) score (Associação Brasileira de Empresas de Pesquisa, 2020). The score is a reliable socioeconomic stratification criterion, classifying socioeconomic status from 0 (lowest economic level) to 100 (highest economic level) and the corresponding average household income. Education level was classified into eight categories (1: illiterate; 2–3: complete-incomplete elementary school; 4–5: complete-incomplete secondary school; 6–7: complete-incomplete college; 8: postgraduate).

### Data analysis

Statistical analyses were performed using IBM SPSS Statistics v. 22.0 (SPSS, Chicago, IL, USA). Data distribution was analyzed using the Shapiro-Wilk test. Continuous variables were described as means and standard deviations or medians and interquartile ranges. Spearman correlation coefficients were used to explore associations with the SUS acceptability score. Significantly correlated (p≥0.05) secondary outcomes were entered in the hierarchical multivariate linear regression analysis to assess their ability to predict the SUS acceptability score. The standardized coefficient (β) of each significant predictor was provided. The structured interviews were analyzed using the qualitative content analysis methodology (de Souza Minayo, 2012), using the inductive method, identifying and coding relevant themes, followed by categories and abstraction arrangement. Unidentified authentic citations exemplified the formulated categories (O'Brien et al., 2014). All p values were two-tailed, with values <0.05 considered statistical significance.

## Results

### Participants' characteristics

Thirty-one participants were included in the study. Table 1 summarises the participants' characteristics. The study sample was primarily women, with mild to moderate COPD (GOLD Stages I and II), with a household economic ABEP score of 15.5 (average US$380.00/month). Most participants had completed secondary school (n=18, 58%), followed by those who had not completed secondary school (n=3, 10%), completed elementary school (n=5, 16%), or did not complete elementary school (n=5, 16%).

**Table 1:** Participants' Characteristics (n=31)

**Age, years**	**63±9**
**Sex, female**	**19 (61.2)**
**BMI, kg/m2**	**27.9±9.18**
**FEV_1_, % predicted**	**73.2±13.2**
**FEV1/FVC**	**63.5±4.4**
**GOLD Stages, n**	
** 1**	**13 (41.9)**
** 2**	**16 (51.6)**
** 3**	**2 (6.4)**
** 4**	**0 (0.0)**
**Pack-years**	**59.4±34.6**
**Active tobacco users, n**	**10 (32.2)**
**Ex-tobacco users, n**	**19 (61.4)**
**Never used tobacco, n**	**2 (6.4)**
**LTOT, n**	**1 (3.0)**
**On systemic corticosteroids, n**	**7 (22.5)**
**CAT**	**20.2±5.3**
**mMRC (0–4)**	**1 (1–2)**
**Exacerbations in the last 12 months**	
** 0**	**23 (74.1)**
** 1**	**5 (16.1)**
** 2**	**1 (3.2)**
** ≥3**	**2 (6.4)**
**Physical Activity**	
** Sedentary time, average/valid days, minutes**	**374±127**
** LPA, average/valid days, minutes**	**827±140**
** MVVPA, average/valid days, minutes**	**19±14**
** Step counts, average/valid days**	**621±348**
**ABEP score**	**15.5 (14.0–18.0)**
**Educational level**	**5 (3–5)**

*Note*. Data are presented as mean±SD, n (%), and median (25th-75th interquartile). Abbreviations: ABEP: Brazilian Association of Research Companies; BMI: Body Mass Index; CAT: Chronic Obstructive Pulmonary Disease Assessment Test; FEV1: Forced Expiratory Volume in 1 second; FVC: Forced Vital Capacity; GOLD: Global Initiative for Chronic Obstructive Lung Disease; LPA: Light Physical Activity; LTOT: on supplemental long-term home oxygen therapy; mMRC: modified Medical Research Council dyspnea scale; MVVPA: Moderate to Very Vigorous Physical Activity; SUS: System Usability Scale.

**Figure 1:**
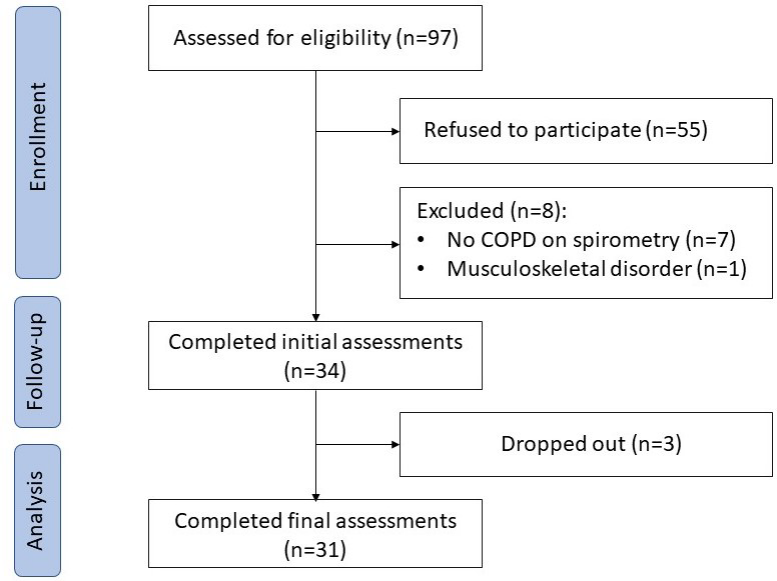
Participant Flow Diagram

The participant flow diagram and reasons for exclusion are shown in Figure 1.

### Feasibility outcomes

**Figure 2:**
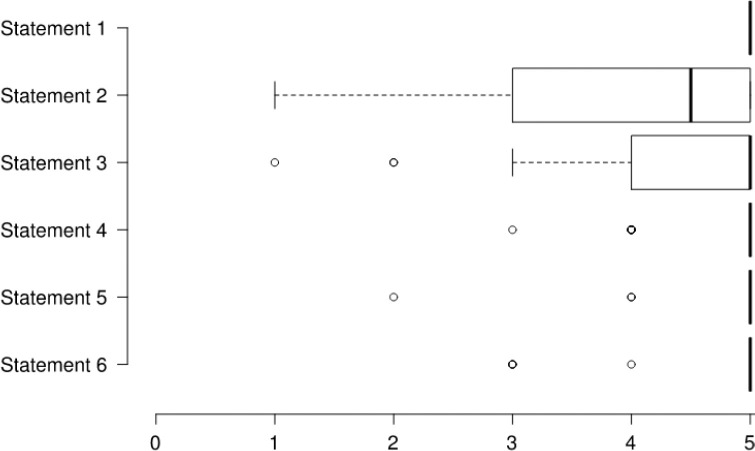
Questionnaire Scores of the Implementation Statements

**Figure 3:**
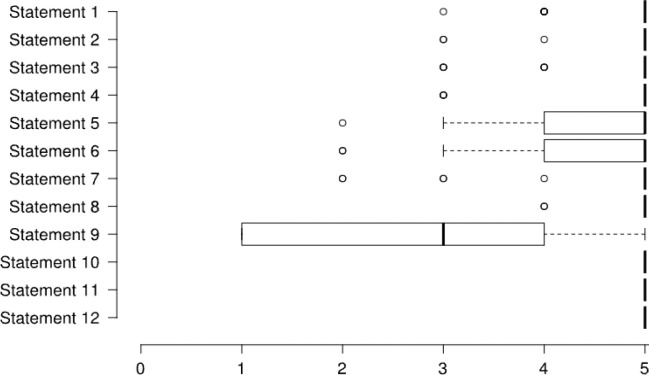
Questionnaire Scores of the Practicality Statements

The median SUS total score for PTR acceptability was 83 (69–95, interquartile range), and twenty-three participants (74.2%) achieved a score of ‘above average’ acceptability (≥68). The mean scores for the implementation and practicality aspects were 4.6±0.3 and 4.5±0.6 out of 5. Figures 2 and 3 demonstrate participants' scores on each statement related to the implementation and practicality aspects of feasibility.

Figure 2 Statement 1: I received all the necessary information about the exercise program during the initial demonstration, Statement 2: This exercise program would interfere with my usual activities, Statement 3: I would like to receive personalised guidance/education through this device, Statement 4: This device would help me exercise at home if I had it available, Statement 5: I would feel more secure while being monitored by the PTR equipment, Statement 6: I would have a specific place available for installing and using these devices at home. Each statement was scored from 1 to 5 (1: totally disagree and 5: totally agree). Centre lines show the medians; box limits indicate the 25th and 75th percentiles; whiskers extend 1.5 times the interquartile range from the 25th and 75th percentiles, and dots represent outliers.

Figure 3 Statement 1: The time spent in the exercise session was adequate, Statement 2: It was easy to report symptoms on exercise, Statement 3: It was easy to use the exercise bike, Statement 4: It was easy to use the oximeter (“finger monitor”), Statement 5: It was easy to use the smartphone, Statement 6: It was easy to find the information on the smartphone, Statement 7: The size of the text displayed on the screen was sufficient, Statement 8: The colors used on the device display were nice, Statement 9: The audiovisual content provided by the smartphone during the session was good, Statement 10: I found the text terms familiar when handling the smartphone, Statement 11: The services offered met my expectations, Statement 12: I would rate/judge the overall service as adequate. Each statement was rated from 1 to 5 (1: totally disagree and 5: totally agree). Centre lines show the medians; box limits indicate the 25th and 75th percentiles; whiskers extend 1.5 times the interquartile range from the 25th and 75th percentiles, and dots represent outliers.

Most participants (n=26, 83.8%) reported no suggested changes related to the PTR adaptation aspect of feasibility. However, the interview identified four categories: an increased variety of exercises (one participant), comfort using equipment (one participant), improved access to PTR (four participants), and financial constraints for equipment acquisition (three participants). Two themes were identified while studying the semantic relationship related to PTR session add-ons, including varying exercise modalities and equipment adaptations to improve comfort while using the exercise bike, as exemplified:

*“… maybe it would help if the exercises include other ways try to do this, including the climbing stairs, to improve the ability to climb in general, right, because you go up the stairs or uphill daily.” (id03)*

*“…the pedal there, that pedal runs off… I think if we could find a way to fasten it better… I think it would work better this way; the person would stop worrying about tiredness here, right? And could ride more smoothly.” (id06)*

And two additional themes related to pulmonary rehabilitation in general, including information on the availability of PTR programs and improvement in access to the service:

*“… In fact, I think that it has to be better advertised because almost nobody knows about it here, but everything was done well here, nothing was missing…” (id22)*

*“…I just think it's too expensive, people with less money wouldn't be able to do it, that's all I think….” (id08)*

No adverse events were registered during the PTR sessions, and all participants completed the simulated endurance and resistance exercise session components.

### Associations Between PTR Acceptability and Secondary Outcomes

The associations between acceptability and secondary outcomes are shown in Table 2. Better acceptability of PTR was associated with lower age, less dyspnea, and higher educational level.

**Table 2:** Associations Between PTR Acceptability on SUS and Secondary Outcomes in Individuals with COPD (n=31)

Secondary outcomes	Spearman correlation coefficient	p-value
Age	-0.57	<0.01
ABEP	0.24	0.19
CAT	-0.22	0.22
mMRC	-0.35	0.056
FEV_1_	0.11	0.56
Physical Activity		
Sedentary time	-0.05	0.77
LPA	-0.22	0.24
MVVPA	0.10	0.60
Step counts	0.14	0.44
Education	0.51	<0.01

*Note*. Abbreviations: ABEP: Brazilian Association of Research Companies; CAT: Chronic Obstructive Pulmonary Disease Assessment Test; FEV1: Forced Expiratory Volume in 1 second; LPA: Light Physical Activity; mMRC: modified Medical Research Council dyspnea scale; MVVPA: Moderate to Very Vigorous Physical Activity; SUS: System Usability Scale.

The multivariate linear regression yielded a significant model which explained 41% of PTR acceptability [R^2^=0.447; Ajusted R^2^=0.41] with age and education level as significant predictors: age (β=-0.425; t=-2.895; p=0.007) and education (β=0.429; t=2.921; p=0.007).

## Discussion

This is the first study to evaluate the PTR feasibility aspects of acceptability, implementation, practicality, and adaptation for individuals with COPD in Brazil. The main findings were: (1) the commercially available and attainable PTR setup is acceptable for this population; (2) the PTR has good practicality and can be implemented as a rehabilitation strategy; (3) the major adaptations required for the PTR were varying exercise modalities and improvement in comfort; and (4) acceptability of PTR for individuals with COPD in Brazil is associated with lower age and higher educational level.

COPD presents with slow clinical progression, aging, reduced muscle mass and exercise tolerance (GOLD, 2022). These factors may contribute to the lower adherence to home-based exercise protocols among older individuals and support our finding that younger participants showed greater acceptability of PTR. More familiarity with the technology used in the simulated session was expected among younger participants. Individuals with advanced age, severe disease, and physical limitations may require in-person support for the initial PTR sessions before independently completing the remaining sessions in a future home-based approach to improve PTR acceptability (King et al., 2020). A higher level of education was also associated with greater acceptability. The education component of pulmonary rehabilitation is crucial (Blackstock & Evans, 2019), and less literacy could affect the delivery and the long-term maintenance of home-based PTR. The learning needs of individuals in PTR should be considered in future research to guide further adaptation requirements for specific learners. Health-related technology education requires understanding of digital and health literacy (Blackstock & Evans, 2019).

This study showed that the PTR has above-average acceptability among Brazilian people with COPD. The commercially available equipment in the country was used, including smartphones and a free video-conferencing application, and equipment commonly used during the exercise component of in-person pulmonary rehabilitation programs in the country (Barreto et al., 2021). The benefits of comprehensive PTR for people with COPD are well documented (Michaelchuk et al., 2022). Good acceptability of a similar PTR setup was also observed in the research conducted in high-income countries (Almojaibel et al., 2021; Holland et al., 2013). In addition, including individuals with mild to moderate COPD at a younger age may have contributed to the greater acceptability observed in our sample. Similar findings were observed by Bonnevie et al. (2021) as younger participants with better exercise capacity presented with more autonomy in remote data transmission during home-based exercise sessions.

There needs to be more data on the implementation and practical aspects of PTR for individuals with COPD (Bedra et al., 2013; Paneroni et al., 2015). Despite varying prior experience with the technology used in this study, most participants reported higher scores on implementation and practicality statements. Feedback from participants provides essential information for developing patient-centered specifications tailored to users' needs, values, and preferences, which can enhance the implementation of PTR in different contexts and cultures, particularly in low-income populations (Bedra et al., 2013; King et al., 2020). In this study, participants indicated some adaptations, including varying exercise modalities and equipment modifications for better comfort. Similar results were obtained in previous investigations, which reported difficulty performing the PTR session and the need for instrument adaptations (Sönnerfors et al, 2020), which is essential for successful technology-based health service provision.

The setup used in the simulated PTR session was safe, with no adverse events registered. This finding suggests that the instruments provided, including a dyspnea scale, commercially available pulse oximetry, and smartphone video conferencing, are suitable to deliver the exercise component of PTR for people with COPD in Brazil. Although only a single laboratory-simulated exercise session was carried out, all participants completed the session. Adequate exercise session attendance (76%) has been reported using a similar home-based PTR setup with a videoconferencing tablet (Holland et al., 2013).

This study identified components in PTR for adequate delivery for Brazilians with COPD. The mixed-methods design explored four feasibility aspects providing an expanded analysis of PTR adaptations required for this population living in a middle-income country context. New opportunities were identified to explore further feasibility aspects among Brazilians, including the demand, integration, and expansion of PTR in the public health system across the country. These findings evidenced the relevant clinical and sociodemographic characteristics of people with COPD who are more likely to accept the PTR intervention in the Brazilian clinical practice and encourage future experimental studies to test the efficacy of PTR in this population.

Some study limitations are to be addressed. This was a cross-sectional study without any longitudinal data. Although the primary purpose of this study was to report the PTR feasibility, further studies are required with longitudinal data on how PTR may affect future exacerbations, respiratory symptoms, and the clinical course of COPD. A convenience sample was recruited, and the feasibility results observed may represent a restricted sample of people with COPD in Brazil. However, the sample's age, education, and income level represent most of the country's population. Since the simulated PTR session was conducted in a controlled environment with regular internet communication, feasibility may differ in a home-based setting. Lastly, questionnaires on practicality and implementation aspects of feasibility were evaluated based on the statements applied in previous investigations of PTR feasibility in COPD (Bedra et al., 2013; Paneroni et al., 2015). Future studies using specifically validated instruments to assess the feasibility of telerehabilitation technologies (Almojaibel et al., 2019) may inform about adequate methods of delivering PTR for Brazilian individuals.

## Conclusions

PTR is safe and feasible for individuals with COPD in Brazil regarding acceptability, implementation, practicality, and adaptation. Younger age and higher educational level are associated with greater PTR acceptability in this population. Further research is required to explore additional aspects of the feasibility and efficacy of PTR for people with COPD in Brazil delivered through home-based sessions.
